# Supplementary material for: Associations of Cooking Salt Intake During Pregnancy with Low Birth Weight and Small for Gestational Age Newborns: A Large Cohort Study
Source: Nutrients. 2025 Feb 11;17(4):642. doi: 10.3390/nu17040642 (PMC11858035; doi:10.3390/nu17040642)
Supplement: Supplementary file 1 [file nutrients-17-00642-s001.zip › nutrients-3432804-supplementary.pdf]

Supplementary Materials

Associations of Cooking Salt Intake During Pregnancy with Low Birth Weight and Small for Gestational Age Newborns: a Large Cohort Study

Supplementary Materials **1**

Tables **3**

Table S1. Baseline characteristics of pregnant women by salt density in Shanghai, China. 3
Table S2. Sensitivity analysis for salt intake and salt density with low birth weight.[a] 5
Table S3. Sensitivity analysis for salt intake and salt density with macrosomia.[a] 8
Table S4. Sensitivity analysis for salt intake and salt density with small for gestational age.[a] 11
Table S5. Sensitivity analysis for salt intake and salt density with large for gestational age.[a] 14
Table S6. Sensitivity analysis for salt intake with birth weight outcomes.[a] 17

Figures **19**

Figure S1. Forest plots for the association of salt intake with macrosomia risk in pre-specified subgroups. 19
Figure S2. Forest plots for the association of salt density with macrosomia risk in pre-specified subgroups. 21
Figure S3. Forest plots for the association of salt intake with the risk of large for gestational age in pre-specified subgroups. 23
Figure S4. Forest plots for the association of salt density with the risk of large for gestational age in pre-specified subgroups. 25

Tables

Table S1. Baseline characteristics of pregnant women by salt density in Shanghai, China.

	Salt density (mg/kcal)				
	**Overall**	**T1 (<1.9)**	**T2 (1.9 to 3.7)**	**T3 (≥3.7)**	**P**
N	4267	1422	1422	1423	
Maternal age, mean (SD), years	29.6 (4.4)	29.9 (4.3)	29.7 (4.3)	29.1 (4.5)	<0.001
Infants sex, male, n (%)	2190 (51.3)	748 (52.6)	726 (51.1)	716 (50.3)	0.461
Maternal domicile location, n (%)					0.491
Northern China	695 (16.3)	224 (15.8)	225 (15.8)	246 (17.3)	
Shanghai surrounding area	2789 (65.4)	950 (66.8)	922 (64.8)	917 (64.4)	
Southern China	783 (18.4)	248 (17.4)	275 (19.3)	260 (18.3)	
Baseline gestational periods, n (%)					0.089
First trimester	1369 (32.1)	472 (33.2)	460 (32.3)	437 (30.7)	
Second trimester	1630 (38.2)	555 (39.0)	511 (35.9)	564 (39.6)	
Third trimester	1268 (29.7)	395 (27.8)	451 (31.7)	422 (29.7)	
Maternal education, <13 years, n (%)	1316 (30.8)	372 (26.2)	420 (29.5)	524 (36.8)	<0.001
Household income, yuan/year, n (%)					<0.001
<100,000	727 (17.0)	195 (13.7)	247 (17.4)	285 (20.0)	
100,000 – 350,000	3041 (71.3)	1035 (72.8)	996 (70.0)	1010 (71.0)	
≥350,000	499 (11.7)	192 (13.5)	179 (12.6)	128 (9.0)	
Pre-pregnancy BMI, kg/m^2, n (%)					0.248
<18.5	553 (13.0)	197 (13.9)	162 (11.4)	194 (13.6)	
18.5-23.9	3002 (70.4)	984 (69.2)	1028 (72.3)	990 (69.6)	
≥24.0	712 (16.7)	241 (16.9)	232 (16.3)	239 (16.8)	
Maternal height, mean (SD), cm	160.9 (5.2)	161.0 (5.2)	160.9 (5.2)	160.8 (5.1)	0.450
Alcohol drinking, never, n (%)	3842 (90.0)	1270 (89.3)	1274 (89.6)	1298 (91.2)	0.187
Passive smoking, never, n (%)	2488 (58.3)	775 (54.5)	860 (60.5)	853 (59.9)	0.002
Leisure-time physical activity, n (%)					0.005
<150 min/week	2347 (55.0)	753 (53.0)	762 (53.6)	832 (58.5)	
≥150 min/week	1920 (45.0)	669 (47.0)	660 (46.4)	591 (41.5)	
Gestational age at birth, mean (SD), weeks	38.9 (1.3)	38.9 (1.3)	38.9 (1.4)	39.0 (1.3)	0.006
Eating at home every day, yes, n (%)	1898 (44.5)	446 (31.4)	650 (45.7)	802 (56.4)	<0.001
Fruits, mean (SD), g [a]	281.4 (226.0)	320.5 (270.6)	280.0 (196.4)	243.8 (196.3)	<0.001
Vegetables, mean (SD), g [a]	221.1 (183.8)	247.8 (212.6)	224.5 (175.0)	190.9 (154.5)	<0.001
Sodium, mean (SD), mg [a]	2804.5 (1432.0)	2814.3 (1407.7)	2804.2 (1395.6)	2795.1 (1491.6)	0.939
Multivitamin, yes, n (%)	1782 (41.8)	613 (43.1)	612 (43.0)	557 (39.1)	0.049
Calcium, yes, n (%)	1752 (41.1)	568 (39.9)	600 (42.2)	584 (41.0)	0.475
Folic acid, yes, n (%)	2636 (61.8)	890 (62.6)	868 (61.0)	878 (61.7)	0.696
Gestational diabetes, yes, n (%)	244 (5.7)	90 (6.3)	71 (5.0)	83 (5.8)	0.300
Pregnancy-induced hypertension, yes, n	106 (2.5)	38 (2.7)	34 (2.4)	34 (2.4)	0.856

(%)					
Nulliparity, yes, n (%)	2482 (58.2)	814 (57.2)	798 (56.1)	870 (61.1)	0.017
Natural labor, yes, n (%)	2251 (52.8)	745 (52.4)	745 (52.4)	761 (53.5)	0.798
Gestational age at delivery, n (%)					0.042
Pre-term	170 (4.0)	62 (4.4)	55 (3.9)	53 (3.7)	
Term	4077 (95.5)	1356 (95.4)	1364 (95.9)	1357 (95.4)	
Post-term	20 (0.5)	4 (0.3)	3 (0.2)	13 (0.9)	

Abbreviations: SD, standard deviation. [a] The intake of fruits, vegetables, and sodium was assessed through the Food Frequency Questionnaire.

Table S2. Sensitivity analysis for salt intake and salt density with low birth weight.[a]

	**Salt intake (g/day)**				**Salt density (mg/kcal)** [b]			
**Variable**	**<5.0**	**5.0-10.0**	**≥10.0**	***P*-trend** [c]	**T1 (<1.9)**	**T2 (1.9 to 3.7)**	**T3 (≥3.7)**	***P*-trend**
**Primary analysis** [d]								
N Cases/Total	47/2122	42/1372	17/539		31/1347	32/1342	43/1344	
Adjusted OR	1.00 (ref)	1.72 (1.01-2.91)	2.06 (1.02-4.13)	0.036	1.00 (ref)	0.93 (0.50-1.73)	1.91 (1.08-3.36)	0.011
**Additional adjustment for fruit and vegetable intake per day** [e]								
N Cases/Total	47/2122	42/1372	17/539		31/1347	32/1342	43/1344	
Adjusted OR	1.00 (ref)	1.73 (1.02-2.93)	2.09 (1.04-4.19)	0.032	1.00 (ref)	0.93 (0.50-1.73)	1.89 (1.07-3.35)	0.012
**Additional adjustment for multivitamin, calcium tablets, and folic acid supplements** [e]								
N Cases/Total	47/2122	42/1372	17/539		31/1347	32/1342	43/1344	
Adjusted OR	1.00 (ref)	1.72 (1.01-2.93)	2.04 (1.01-4.10)	0.039	1.00 (ref)	0.92 (0.50-1.71)	1.89 (1.07-3.34)	0.012
**Additional adjustment for gestational diabetes at baseline** [e]								
N Cases/Total	47/2122	42/1372	17/539		31/1347	32/1342	43/1344	
Adjusted OR	1.00 (ref)	1.72 (1.01-2.92)	2.08 (1.03-4.19)	0.033	1.00 (ref)	0.93 (0.50-1.72)	1.92 (1.09-3.37)	0.010
**Additional adjustment for sodium from food** [e]								
N Cases/Total	47/2122	42/1372	17/539		31/1347	32/1342	43/1344	

	Salt intake (g/day)				Salt density (mg/kcal) [b]			
**Variable**	**<5.0**	**5.0-10.0**	**≥10.0**	***P*-trend** [c]	**T1 (<1.9)**	**T2 (1.9 to 3.7)**	**T3 (≥3.7)**	***P*-trend**
Adjusted OR	1.00 (ref)	1.72 (1.01-2.92)	2.08 (1.03-4.18)	0.035	1.00 (ref)	0.95 (0.51-1.77)	2.00 (1.12-3.56)	0.008
**Additional adjustment for nulliparity** [e]								
N Cases/Total	47/2122	42/1372	17/539		31/1347	32/1342	43/1344	
Adjusted OR	1.00 (ref)	1.72 (1.01-2.94)	2.02 (1.00-4.08)	0.0424	1.00 (ref)	0.91 (0.48-1.69)	1.83 (1.03-3.25)	0.0158
**Excluding participants with pregnancy-induced hypertension at baseline** [d]								
N Cases/Total	46/2068	40/1333	16/523		31/1310	30/1307	41/1307	
Adjusted OR	1.00 (ref)	1.71 (1.00-2.93)	2.10 (1.04-4.23)	0.033	1.00 (ref)	0.90 (0.48-1.69)	1.89 (1.07-3.33)	0.012
**Including participants whose infants born in term (born between 37 to <42 completed weeks)** [d]								
N Cases/Total	13/2019	17/1311	10/517		11/1282	9/1285	20/1280	
Adjusted OR	1.00 (ref)	1.98 (0.92-4.25)	2.75 (1.13-6.72)	0.030	1.00 (ref)	0.84 (0.34-2.08)	1.97 (0.90-4.32)	0.042
**Including participants with spontaneous labors** [d]								
N Cases/Total	20/1151	20/710	7/303		14/723	14/710	19/731	
Adjusted OR	1.00 (ref)	2.36 (1.06-5.28)	2.14 (0.72-6.37)	0.147	1.00 (ref)	1.10 (0.43-2.85)	1.93 (0.80-4.64)	0.108
**Handling missing covariate values using multiple imputation** [d]								
N Cases/Total	47/2122	42/1372	17/539		31/1347	32/1342	43/1344	
Adjusted OR	1.00 (ref)	1.68 (0.99-2.85)	1.98 (0.99-3.98)	0.047	1.00 (ref)	0.92 (0.5-1.72)	1.86 (1.06-3.29)	0.013

Abbreviations: N Cases/Total=number of cases and total participants in the study. OR=odds ratio. CI=confidence interval. Ref=reference. *P*-trend=*P* value for trend.

[a] ORs and 95% CIs for the association were calculated in logistic models.

[b] The first and second tertiles of salt density in the population were 1.9 and 3.7 mg/kcal, respectively.

[c] The *P* values for trend were obtained through modelling the median value of each category into the logistic regression models.

[d] Covariates adjusted in the models included maternal age (continuous variables, in years), infant sex (male or female), maternal domicile (south China, north China, or surrounding area of Shanghai), gestational group at recruitment (first, second, or third trimester), maternal education (<13 or ≥13 years), annual family income (<100 000, 100 000-350 000, or ≥350 000 yuan/year), pre-pregnancy BMI (<18.5, 18.5-23.9, or ≥24.0 kg/m^2), height (continuous variables, in cm), passive smoking during pregnancy (never or ever), alcohol drinking (never or ever), physical activity (regular physical activity or not), eating at home every day (yes or no), energy intake per day (continuous variables, in kcal/day) and gestational week at birth (continuous variables, in weeks). Adjustment for energy intake was no longer applied in the analysis of salt density.

[e] Covariates adjusted in the models were based on the those included in the primary analysis.

Table S3. Sensitivity analysis for salt intake and salt density with macrosomia.[a]

	Salt intake (g/day)				Salt density (mg/kcal) [b]			
**Variable**	**<5.0**	**5.0-10.0**	**≥10.0**	***P*-trend** [c]	**T1 (<1.9)**	**T2 (1.9 to 3.7)**	**T3 (≥3.7)**	***P*-trend**
**Primary analysis** [d]								
N Cases/Total	106/2181	93/1423	35/557		75/1391	80/1390	79/1380	
Adjusted OR	1.00 (ref)	1.29 (0.96-1.75)	1.22 (0.80-1.85)	0.299	1.00 (ref)	1.05 (0.76-1.47)	0.98 (0.69-1.38)	0.834
**Additional adjustment for fruit and vegetable intake per day** [e]								
N Cases/Total	106/2181	93/1423	35/557		75/1391	80/1390	79/1380	
Adjusted OR	1.00 (ref)	1.30 (0.96-1.76)	1.23 (0.81-1.86)	0.281	1.00 (ref)	1.08 (0.77-1.51)	1.02 (0.72-1.45)	0.965
**Additional adjustment for multivitamin, calcium tablets, and folic acid supplements** [e]								
N Cases/Total	106/2181	93/1423	35/557		75/1391	80/1390	79/1380	
Adjusted OR	1.00 (ref)	1.30 (0.96-1.76)	1.22 (0.80-1.85)	0.297	1.00 (ref)	1.06 (0.76-1.48)	0.98 (0.70-1.39)	0.867
**Additional adjustment for gestational diabetes at baseline** [e]								
N Cases/Total	106/2181	93/1423	35/557		75/1391	80/1390	79/1380	
Adjusted OR	1.00 (ref)	1.29 (0.95-1.74)	1.21 (0.80-1.84)	0.312	1.00 (ref)	1.07 (0.76-1.49)	0.98 (0.70-1.38)	0.847
**Additional adjustment for sodium from food at baseline** [e]								
N Cases/Total	106/2181	93/1423	35/557		75/1391	80/1390	79/1380	
Adjusted OR	1.00 (ref)	1.29 (0.95-1.75)	1.21 (0.80-1.83)	0.320	1.00 (ref)	1.06 (0.75-1.48)	0.98 (0.69-1.39)	0.845
**Additional adjustment for nulliparity** [e]								
N Cases/Total	106/2181	93/1423	35/557		75/1391	80/1390	79/1380	
Adjusted OR	1.00 (ref)	1.30 (0.96-1.76)	1.23 (0.81-1.87)	0.274	1.00 (ref)	1.06 (0.76-1.48)	0.99 (0.70-1.39)	0.894
**Excluding participants with pregnancy-induced hypertension at baseline** [d]								
N Cases/Total	103/2125	91/1384	34/541		73/1352	78/1355	77/1343	

	Salt intake (g/day)				Salt density (mg/kcal) [b]			
**Variable**	**<5.0**	**5.0-10.0**	**≥10.0**	***P*-trend** [c]	**T1 (<1.9)**	**T2 (1.9 to 3.7)**	**T3 (≥3.7)**	***P*-trend**
Adjusted OR	1.00 (ref)	1.30 (0.95-1.76)	1.21 (0.79-1.84)	0.326	1.00 (ref)	1.04 (0.74-1.47)	0.97 (0.69-1.37)	0.805
**Including participants whose infants born in term (born between 37 to <42 completed weeks)** [d]								
N Cases/Total	105/2111	91/1385	34/541		74/1345	79/1355	77/1337	
Adjusted OR	1.00 (ref)	1.26 (0.93-1.72)	1.22 (0.80-1.85)	0.308	1.00 (ref)	1.05 (0.75-1.46)	0.98 (0.69-1.38)	0.838
**Including participants with spontaneous labors** [d]								
N Cases/Total	37/1168	36/726	12/308		22/731	34/730	29/741	
Adjusted OR	1.00 (ref)	1.34 (0.81-2.21)	1.08 (0.54-2.18)	0.760	1.00 (ref)	1.45 (0.82-2.55)	1.17 (0.64-2.11)	0.825
**Handling missing covariate values using multiple imputation** [d]								
N Cases/Total	106/2181	93/1423	35/557		75/1391	80/1390	79/1380	
Adjusted OR	1.00 (ref)	1.29 (0.95-1.75)	1.22 (0.8-1.85)	0.299	1.00 (ref)	1.06 (0.76-1.47)	0.98 (0.7-1.38)	0.846

Abbreviations: N Cases/Total=number of cases and total participants in the study. OR=odds ratio. CI=confidence interval. Ref=reference. *P*-trend=*P* value for trend.

[a] ORs and 95% CIs for the association were calculated in logistic models.

[b] The first and second tertiles of salt density in the population were 1.9 and 3.7 mg/kcal, respectively.

[c] The *P* values for trend were obtained through modelling the median value of each category into the logistic regression models.

[d] Covariates adjusted in the models included maternal age (continuous variables, in years), infant sex (male or female), maternal domicile (south China, north China, or surrounding area of Shanghai), gestational group at recruitment (first, second, or third

trimester), maternal education (<13 or ≥13 years), annual family income (<100 000, 100 000-350 000, or ≥350 000 yuan/year), pre-pregnancy BMI (<18.5, 18.5-23.9, or ≥24.0 kg/m^2), height (continuous variables, in cm), passive smoking during pregnancy (never or ever), alcohol drinking (never or ever), physical activity (regular physical activity or not), eating at home every day (yes or no), energy intake per day (continuous variables, in kcal/day) and gestational week at birth (continuous variables, in weeks). Adjustment for energy intake was no longer applied in the analysis of salt density.

[e] Covariates adjusted in the models were based on the those included in the primary analysis.

Table S4. Sensitivity analysis for salt intake and salt density with small for gestational age.[a]

	Salt intake (g/day)				Salt density (mg/kcal) [b]			
**Variable**	**<5.0**	**5.0-10.0**	**≥10.0**	***P*-trend** [c]	**T1 (<1.9)**	**T2 (1.9 to 3.7)**	**T3 (≥3.7)**	***P*-trend**
**Primary analysis** [d]								
N Cases/Total	105/1861	99/1191	46/478		68/1174	67/1176	115/1180	
Adjusted OR	1.00 (ref)	1.46 (1.09-1.97)	1.69 (1.16-2.47)	0.006	1.00 (ref)	0.96 (0.68-1.37)	1.63 (1.18-2.25)	0.001
**Additional adjustment for fruit and vegetable intake per day** [e]								
N Cases/Total	105/1861	99/1191	46/478		68/1174	67/1176	115/1180	
Adjusted OR	1.00 (ref)	1.47 (1.09-1.98)	1.70 (1.16-2.49)	0.005	1.00 (ref)	0.97 (0.68-1.39)	1.66 (1.19-2.31)	0.001
**Additional adjustment for multivitamin, calcium tablets, and folic acid supplements** [e]								
N Cases/Total	105/1861	99/1191	46/478		68/1174	67/1176	115/1180	
Adjusted OR	1.00 (ref)	1.46 (1.09-1.97)	1.67 (1.14-2.45)	0.007	1.00 (ref)	0.96 (0.67-1.37)	1.63 (1.18-2.25)	0.001
**Additional adjustment for gestational diabetes at baseline** [e]								
N Cases/Total	105/1861	99/1191	46/478		68/1174	67/1176	115/1180	
Adjusted OR	1.00 (ref)	1.47 (1.09-1.97)	1.70 (1.16-2.48)	0.005	1.00 (ref)	0.96 (0.67-1.37)	1.63 (1.18-2.26)	0.001
**Additional adjustment for sodium from food at baseline** [e]								
N Cases/Total	105/1861	99/1191	46/478		68/1174	67/1176	115/1180	
Adjusted OR	1.00 (ref)	1.45 (1.08-1.96)	1.67 (1.14-2.45)	0.007	1.00 (ref)	0.95 (0.67-1.36)	1.60 (1.15-2.23)	0.001
**Additional adjustment for nulliparity** [e]								
N Cases/Total	105/1861	99/1191	46/478		68/1174	67/1176	115/1180	

	Salt intake (g/day)				Salt density (mg/kcal) [b]			
**Variable**	**<5.0**	**5.0-10.0**	**≥10.0**	***P*-trend** [c]	**T1 (<1.9)**	**T2 (1.9 to 3.7)**	**T3 (≥3.7)**	***P*-trend**
Adjusted OR	1.00 (ref)	1.46 (1.08-1.97)	1.66 (1.13-2.42)	0.008	1.00 (ref)	0.96 (0.68-1.37)	1.60 (1.15-2.21)	0.001
**Excluding participants with pregnancy-induced hypertension at baseline** [d]								
N Cases/Total	104/1821	97/1157	45/463		67/1145	67/1148	112/1148	
Adjusted OR	1.00 (ref)	1.46 (1.09-1.98)	1.71 (1.17-2.51)	0.005	1.00 (ref)	0.99 (0.69-1.41)	1.65 (1.19-2.28)	0.001
**Including participants whose infants born in term (born between 37 to <42 completed weeks)** [d]								
N Cases/Total	99/1780	93/1142	41/461		64/1125	64/1129	105/1129	
Adjusted OR	1.00 (ref)	1.44 (1.06-1.96)	1.55 (1.04-2.31)	0.027	1.00 (ref)	0.98 (0.68-1.41)	1.58 (1.13-2.21)	0.002
**Including participants with spontaneous labors** [d]								
N Cases/Total	54/1040	66/638	26/272		30/645	44/642	72/663	
Adjusted OR	1.00 (ref)	2.14 (1.44-3.17)	1.87 (1.12-3.12)	0.015	1.00 (ref)	1.62 (0.99-2.63)	2.47 (1.56-3.91)	<0.0001
**Handling missing covariate values using multiple imputation** [d]								
N Cases/Total	105/1861	99/1191	46/478		68/1174	67/1176	115/1180	
Adjusted OR	1.00 (ref)	1.45 (1.08-1.95)	1.68 (1.14-2.45)	0.007	1.00 (ref)	0.96 (0.67-1.36)	1.62 (1.17-2.24)	0.001

Abbreviations: N Cases/Total=number of cases and total participants in the study. OR=odds ratio. CI=confidence interval.

Ref=reference. *P*-trend=*P* value for trend.

[a] ORs and 95% CIs for the association were calculated in logistic models.

[b] The first and second tertiles of salt density in the population were 1.9 and 3.7 mg/kcal, respectively.

[c] The *P* values for trend were obtained through modelling the median value of each category into the logistic regression models.

[d] Covariates adjusted in the models included maternal age (continuous variables, in years), infant sex (male or female), maternal domicile (south China, north China, or surrounding area of Shanghai), gestational group at recruitment (first, second, or third trimester), maternal education (<13 or ≥13 years), annual family income (<100 000, 100 000-350 000, or ≥350 000 yuan/year), pre-pregnancy BMI (<18.5, 18.5-23.9, or ≥24.0 kg/m^2), height (continuous variables, in cm), passive smoking during pregnancy (never or ever), alcohol drinking (never or ever), physical activity (regular physical activity or not), eating at home every day (yes or no), and energy intake per day (continuous variables, in kcal/day). Adjustment for energy intake was no longer applied in the analysis of salt density.

[e] Covariates adjusted in the models were based on the those included in the primary analysis.

Table S5. Sensitivity analysis for salt intake and salt density with large for gestational age.[a]

	Salt intake (g/day)				Salt density (mg/kcal) [b]			
**Variable**	**<5.0**	**5.0-10.0**	**≥10.0**	***P*-trend** [c]	**T1 (<1.9)**	**T2 (1.9 to 3.7)**	**T3 (≥3.7)**	***P*-trend**
**Primary analysis** [d]								
N Cases/Total	367/2123	274/1366	94/526		248/1354	246/1355	241/1306	
Adjusted OR	1.00 (ref)	1.15 (0.96-1.38)	0.98 (0.75-1.27)	0.972	1.00 (ref)	0.98 (0.80-1.20)	0.99 (0.81-1.22)	0.978
**Additional adjustment for fruit and vegetable intake per day** [e]								
N Cases/Total	367/2123	274/1366	94/526		248/1354	246/1355	241/1306	
Adjusted OR	1.00 (ref)	1.16 (0.96-1.39)	0.98 (0.75-1.27)	0.972	1.00 (ref)	0.99 (0.81-1.22)	1.02 (0.82-1.25)	0.861
**Additional adjustment for multivitamin, calcium tablets, and folic acid supplements** [e]								
N Cases/Total	367/2123	274/1366	94/526		248/1354	246/1355	241/1306	
Adjusted OR	1.00 (ref)	1.15 (0.96-1.39)	0.99 (0.76-1.28)	0.933	1.00 (ref)	0.98 (0.81-1.20)	0.99 (0.81-1.22)	0.973
**Additional adjustment for gestational diabetes at baseline** [e]								
N Cases/Total	367/2123	274/1366	94/526		248/1354	246/1355	241/1306	
Adjusted OR	1.00 (ref)	1.15 (0.96-1.38)	0.97 (0.75-1.26)	0.989	1.00 (ref)	0.99 (0.81-1.21)	1.00 (0.81-1.22)	0.989
**Additional adjustment for sodium from food at baseline** [e]								
N Cases/Total	367/2123	274/1366	94/526		248/1354	246/1355	241/1306	
Adjusted OR	1.00 (ref)	1.15 (0.96-1.38)	0.97 (0.75-1.26)	0.979	1.00 (ref)	0.98 (0.80-1.20)	1.00 (0.81-1.23)	0.991
**Additional adjustment for nulliparity** [e]								
N Cases/Total	367/2123	274/1366	94/526		248/1354	246/1355	241/1306	

	Salt intake (g/day)				Salt density (mg/kcal) [b]			
**Variable**	**<5.0**	**5.0-10.0**	**≥10.0**	***P*-trend** [c]	**T1 (<1.9)**	**T2 (1.9 to 3.7)**	**T3 (≥3.7)**	***P*-trend**
Adjusted OR	1.00 (ref)	1.15 (0.96-1.39)	1.00 (0.77-1.30)	0.830	1.00 (ref)	0.98 (0.80-1.20)	1.02 (0.83-1.25)	0.843
**Excluding participants with pregnancy-induced hypertension at baseline** [d]								
N Cases/Total	350/2067	267/1327	92/510		238/1316	237/1318	234/1270	
Adjusted OR	1.00 (ref)	1.18 (0.98-1.43)	1.01 (0.77-1.31)	0.786	1.00 (ref)	0.98 (0.80-1.21)	1.00 (0.81-1.23)	0.96
**Including participants whose infants born in term (born between 37 to <42 completed weeks)** [d]								
N Cases/Total	344/2025	260/1309	90/510		231/1292	235/1300	228/1252	
Adjusted OR	1.00 (ref)	1.17 (0.97-1.42)	0.99 (0.76-1.30)	0.872	1.00 (ref)	1.02 (0.83-1.25)	1.02 (0.83-1.26)	0.853
**Including participants with spontaneous labors** [d]								
N Cases/Total	148/1134	108/680	42/288		100/715	102/700	96/687	
Adjusted OR	1.00 (ref)	1.20 (0.90-1.59)	1.11 (0.75-1.64)	0.516	1.00 (ref)	1.00 (0.73-1.36)	1.00 (0.73-1.37)	0.980
**Handling missing covariate values using multiple imputation** [d]								
N Cases/Total	367/2123	274/1366	94/526		248/1354	246/1355	241/1306	
Adjusted OR	1.00 (ref)	1.15 (0.96-1.38)	0.97 (0.75-1.27)	0.996	1.00 (ref)	0.98 (0.8-1.2)	0.99 (0.81-1.22)	0.962

Abbreviations: N Cases/Total=number of cases and total participants in the study. OR=odds ratio. CI=confidence interval.

Ref=reference. *P*-trend=*P* value for trend.

[a] ORs and 95% CIs for the association were calculated in logistic models.

[b] The first and second tertiles of salt density in the population were 1.9 and 3.7 mg/kcal, respectively.

[c] The *P* values for trend were obtained through modelling the median value of each category into the logistic regression models.

[d] Covariates adjusted in the models included maternal age (continuous variables, in years), infant sex (male or female), maternal domicile (south China, north China, or surrounding area of Shanghai), gestational group at recruitment (first, second, or third trimester), maternal education (<13 or ≥13 years), annual family income (<100 000, 100 000-350 000, or ≥350 000 yuan/year), pre-pregnancy BMI (<18.5, 18.5-23.9, or ≥24.0 kg/m^2), height (continuous variables, in cm), passive smoking during pregnancy (never or ever), alcohol drinking (never or ever), physical activity (regular physical activity or not), eating at home every day (yes or no), and energy intake per day (continuous variables, in kcal/day). Adjustment for energy intake was no longer applied in the analysis of salt density.

[e] Covariates adjusted in the models were based on the those included in the primary analysis.

Table S6. Sensitivity analysis for salt intake with birth weight outcomes.[a]

	**Salt intake (g/day)**			
**Outcome**	**T1 (<3.5)**	**T2 (3.5 to 6.2)**	**T3 (≥6.2)**	***P*-trend** [b]
**Low birth weight**				
N cases/Total	30/1348	29/1349	47/1336	
Adjusted OR [c]	1.00 (ref)	1.36 (0.73-2.51)	2.22 (1.21-4.06)	0.008
**Small for gestational age**				
N cases/Total	69/1175	71/1197	110/1158	
Adjusted OR [c]	1.00 (ref)	0.98 (0.69-1.39)	1.59 (1.14-2.22)	0.002
**Macrosomia**				
N cases/Total	74/1392	73/1393	87/1376	
Adjusted OR [c]	1.00 (ref)	0.90 (0.64-1.27)	1.07 (0.76-1.51)	0.600
**Large for gestational age**				
N cases/Total	247/1353	225/1351	263/1311	
Adjusted OR [c]	1.00 (ref)	0.86 (0.70-1.06)	1.06 (0.86-1.30)	0.403

Abbreviations: N Cases/Total=number of cases and total participants in the study. OR=odds ratio. CI=confidence interval. Ref=reference. *P*-trend=*P* value for trend.

[a] ORs and 95% CIs for the association were calculated in logistic models.

[b] The *P* values for trend were obtained through modelling the median value of each category into the logistic regression models.

[c] Covariates adjusted in the models included maternal age (continuous variables, in years), infant sex (male or female), maternal domicile (south China, north China, or surrounding area of Shanghai), gestational group at recruitment (first, second, or third trimester), maternal education (<13 or ≥13 years), annual family income (<100 000, 100 000-350 000, or ≥350 000

yuan/year), pre-pregnancy BMI (<18.5, 18.5-23.9, or ≥24.0 kg/m^2), height (continuous variables, in cm), passive smoking during pregnancy (never or ever), alcohol drinking (never or ever), physical activity (regular physical activity or not), eating at home every day (yes or no), and energy intake per day (continuous variables, in kcal/day). Adjustment for energy intake was no longer applied in the analysis of salt density. Analysis for low birth weight and macrosomia were additionally adjusted for gestational week at birth in the final model.

Figures

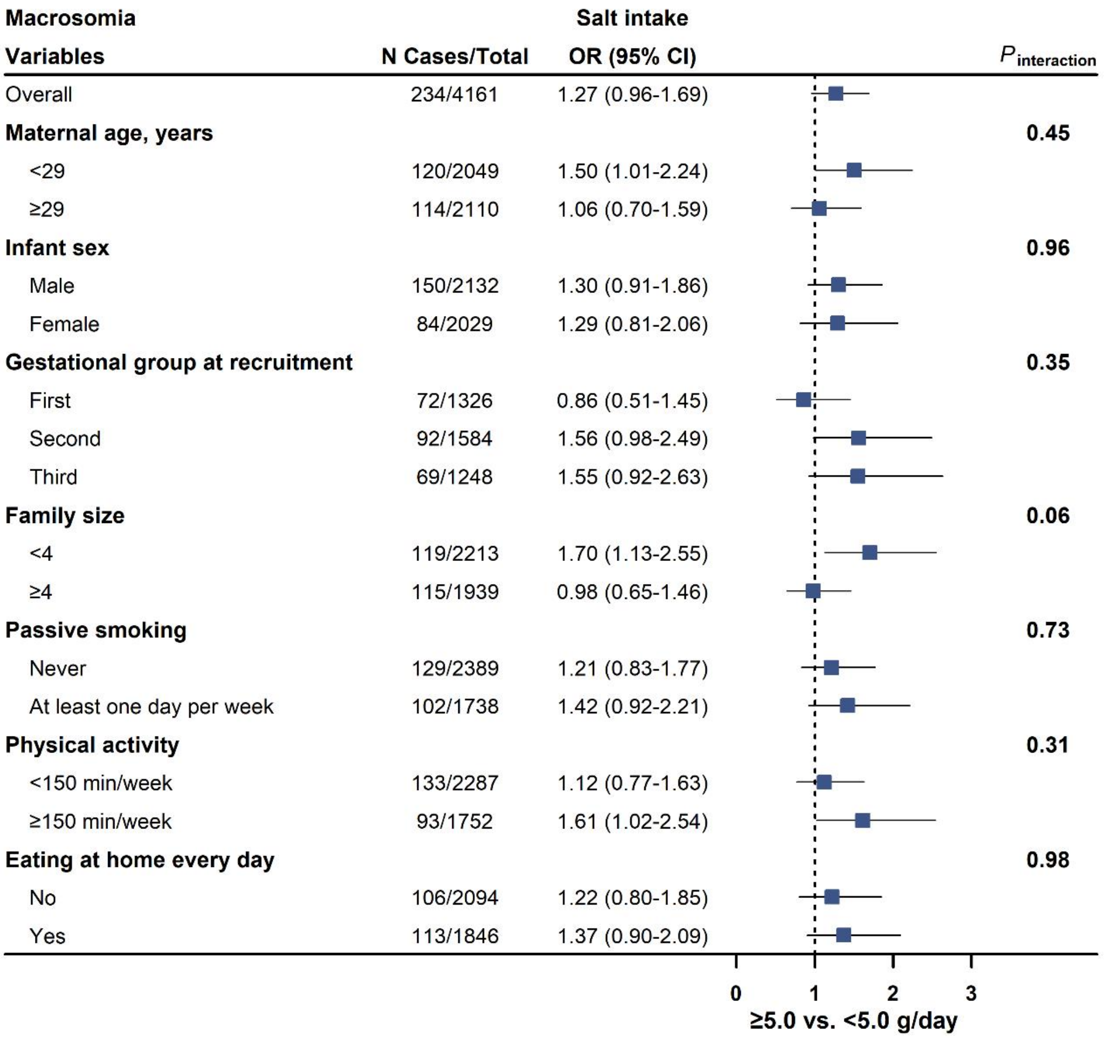

Figure S1. Forest plots for the association of salt intake with macrosomia risk in pre-specified subgroups.

ORs and 95% CI were calculated in logistic model adjusted for maternal age (continuous variables, in years), infant sex (male or female), maternal domicile (south China, north China, or surrounding area of Shanghai), gestational group at recruitment (first, second, or third trimester), maternal education (<13 years or ≥13 years), annual family income (<100 000, 100 000-350 000, or ≥350 000 yuan/year), pre-pregnancy BMI (<18.5, 18.5-23.9, or ≥24.0 kg/m^2),

height (continuous variables, in cm), passive smoking during pregnancy (never or ever), alcohol drinking (never or ever), physical activity (regular physical activity or not), eating at home every day (yes or no), energy intake per day (continuous variables, in kcal/day) and gestational week at birth (continuous variables, in weeks). Salt intake was categorized into two groups (<5.0 (ref) and ≥5.0 g/day) to minimize the small sample bias and improve the precision of estimates. N Cases/Total=number of cases and total participants in the study. OR=odds ratio. CI=confidence interval.

Macrosomia Variables	N Cases/Total	Salt density OR (95% CI)	$P_{interaction}$
Overall	234/4161	0.94 (0.71-1.26)	
**Maternal age, years**			**0.74**
<29	120/2049	0.95 (0.64-1.41)	
≥29	114/2110	0.96 (0.63-1.46)	
**Infant sex**			**0.73**
Male	150/2132	0.92 (0.64-1.33)	
Female	84/2029	1.04 (0.65-1.66)	
**Gestational group at recruitment**			**0.69**
First	72/1326	0.92 (0.55-1.56)	
Second	92/1584	1.06 (0.67-1.68)	
Third	69/1248	0.90 (0.52-1.56)	
**Family size**			**0.09**
<4	119/2213	1.21 (0.82-1.78)	
≥4	115/1939	0.80 (0.54-1.19)	
**Passive smoking**			**0.54**
Never	129/2389	1.01 (0.69-1.48)	
At least one day per week	102/1738	0.90 (0.57-1.42)	
**Physical activity**			**0.42**
<150 min/week	133/2287	1.03 (0.71-1.50)	
≥150 min/week	93/1752	0.86 (0.54-1.38)	
**Eating at home every day**			**0.05**
No	106/2094	0.65 (0.40-1.08)	
Yes	113/1846	1.23 (0.82-1.82)	

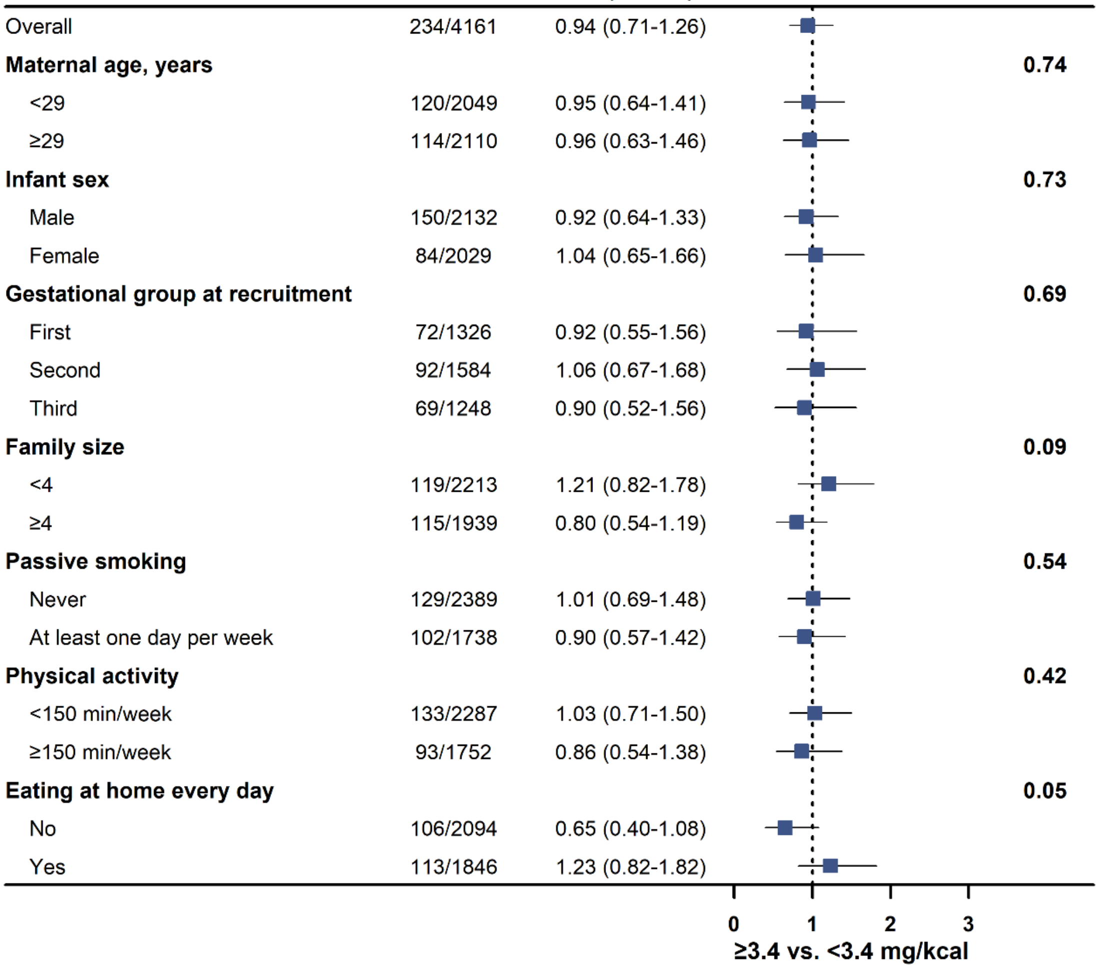

Figure S2. Forest plots for the association of salt density with macrosomia risk in pre-specified subgroups.

ORs and 95% CI were calculated in logistic model adjusted for maternal age (continuous variables, in years), infant sex (male or female), maternal domicile (south China, north China, or surrounding area of Shanghai), gestational group at recruitment (first, second, or third trimester), maternal education (<13 years or ≥13 years), annual family income (<100 000¥, 100 000-350 000, or ≥350 000 yuan/year), pre-pregnancy BMI (<18.5, 18.5-23.9, or ≥24.0 kg/m^2), height (continuous variables, in cm), passive smoking during pregnancy

(never or ever), alcohol drinking (never or ever), physical activity (regular physical activity or not), eating at home every day (yes or no), and gestational week at birth (continuous variables, in weeks). Salt density was categorized into two groups using the mean values (<3.4 (ref) and ≥3.4 mg/kcal) to minimize the small sample bias and improve the precision of estimates. N Cases/Total=number of cases and total participants in the study. OR=odds ratio. CI=confidence interval.

Large for gestational age		Salt intake	
Variables	N Cases/Total	OR (95% CI)	$P_{interaction}$
Overall	735/4015	1.10 (0.93-1.31)	
**Maternal age, years**			**0.34**
<29	331/1944	1.24 (0.96-1.60)	
≥29	404/2069	0.99 (0.78-1.25)	
**Infant sex**			**0.31**
Male	358/2049	1.20 (0.94-1.53)	
Female	377/1966	1.03 (0.82-1.31)	
**Gestational group at recruitment**			**0.94**
First	244/1293	1.09 (0.81-1.48)	
Second	291/1526	1.09 (0.83-1.43)	
Third	199/1193	1.23 (0.88-1.70)	
**Family size**			**0.81**
<4	383/2136	1.07 (0.84-1.35)	
≥4	351/1870	1.17 (0.91-1.51)	
**Passive smoking**			**0.27**
Never	407/2298	1.04 (0.83-1.31)	
At least one day per week	323/1683	1.20 (0.92-1.56)	
**Physical activity**			**0.66**
<150 min/week	410/2206	1.11 (0.88-1.39)	
≥150 min/week	306/1688	1.06 (0.81-1.38)	
**Eating at home every day**			**0.67**
No	348/2032	1.00 (0.78-1.29)	
Yes	341/1771	1.08 (0.84-1.39)	

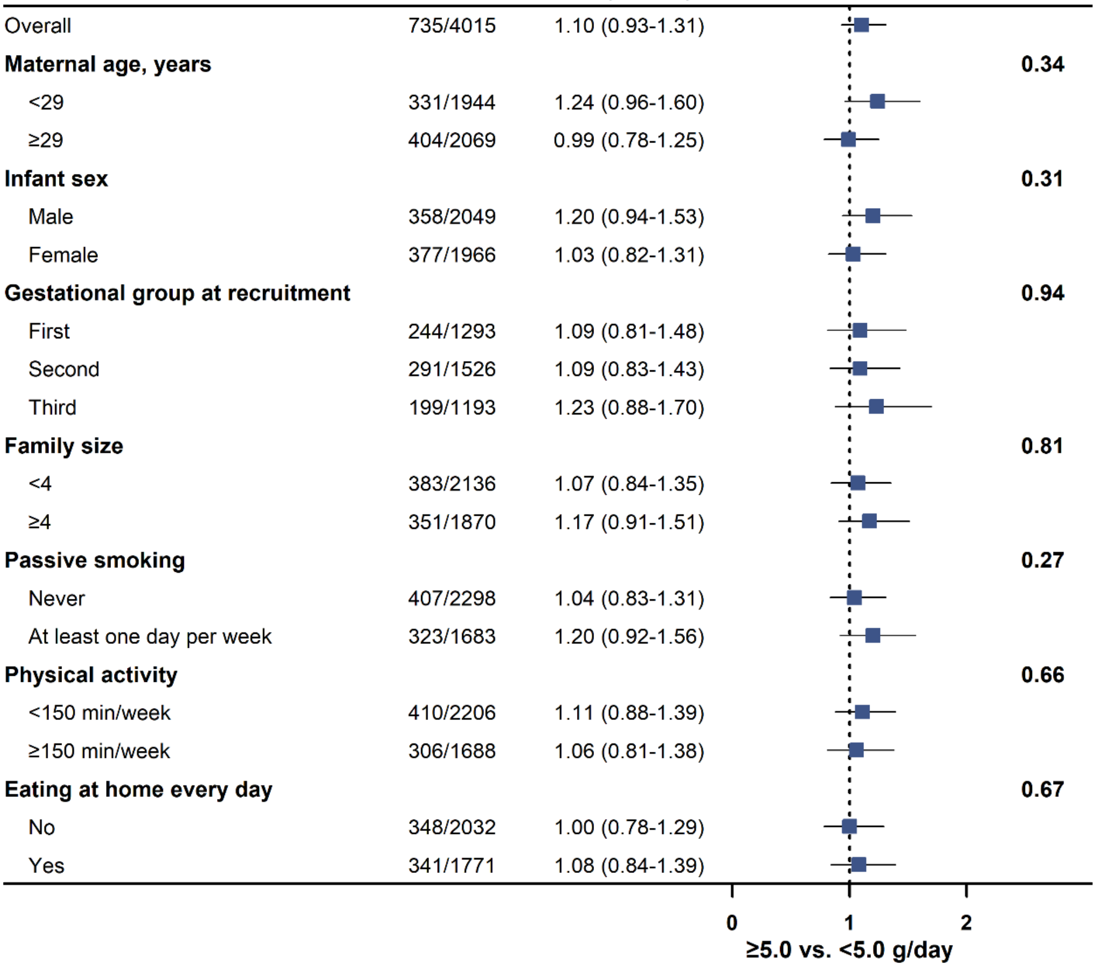

Figure S3. Forest plots for the association of salt intake with the risk of large for gestational age in pre-specified subgroups.

ORs and 95% CI were calculated in logistic model adjusted for maternal age (continuous variables, in years), infant sex (male or female), maternal domicile (south China, north China, or surrounding area of Shanghai), gestational group at recruitment (first, second, or third trimester), maternal education (<13 years or ≥13 years), annual family income (<100 000, 100 000-350 000, or ≥350 000 yuan/year), pre-pregnancy BMI (<18.5, 18.5-23.9, or ≥24.0 kg/m^2), height (continuous variables, in cm), passive smoking during pregnancy

(never or ever), alcohol drinking (never or ever), physical activity (regular physical activity or not), eating at home every day (yes or no), and energy intake per day (continuous variables, in kcal/day). Salt intake was categorized into two groups (<5.0 (ref) and ≥5.0 g/day) to minimize the small sample bias and improve the precision of estimates. N Cases/Total=number of cases and total participants in the study. OR=odds ratio. CI=confidence interval.

Large for gestational age		Salt density	
Variables	N Cases/Total	OR (95% CI)	$P_{interaction}$
Overall	735/4015	0.94 (0.79-1.12)	
**Maternal age, years**			**0.97**
<29	331/1944	0.97 (0.76-1.25)	
≥29	404/2069	0.91 (0.71-1.16)	
**Infant sex**			**0.60**
Male	358/2049	0.97 (0.76-1.25)	
Female	377/1966	0.92 (0.72-1.17)	
**Gestational group at recruitment**			**0.69**
First	244/1293	1.01 (0.74-1.37)	
Second	291/1526	0.89 (0.67-1.18)	
Third	199/1193	0.98 (0.70-1.37)	
**Family size**			**0.50**
<4	383/2136	0.87 (0.69-1.10)	
≥4	351/1870	1.03 (0.81-1.31)	
**Passive smoking**			**0.73**
Never	407/2298	0.98 (0.78-1.24)	
At least one day per week	323/1683	0.89 (0.68-1.17)	
**Physical activity**			**0.15**
<150 min/week	410/2206	1.01 (0.80-1.27)	
≥150 min/week	306/1688	0.79 (0.60-1.05)	
**Eating at home every day**			**0.77**
No	348/2032	0.87 (0.66-1.14)	
Yes	341/1771	0.88 (0.69-1.13)	

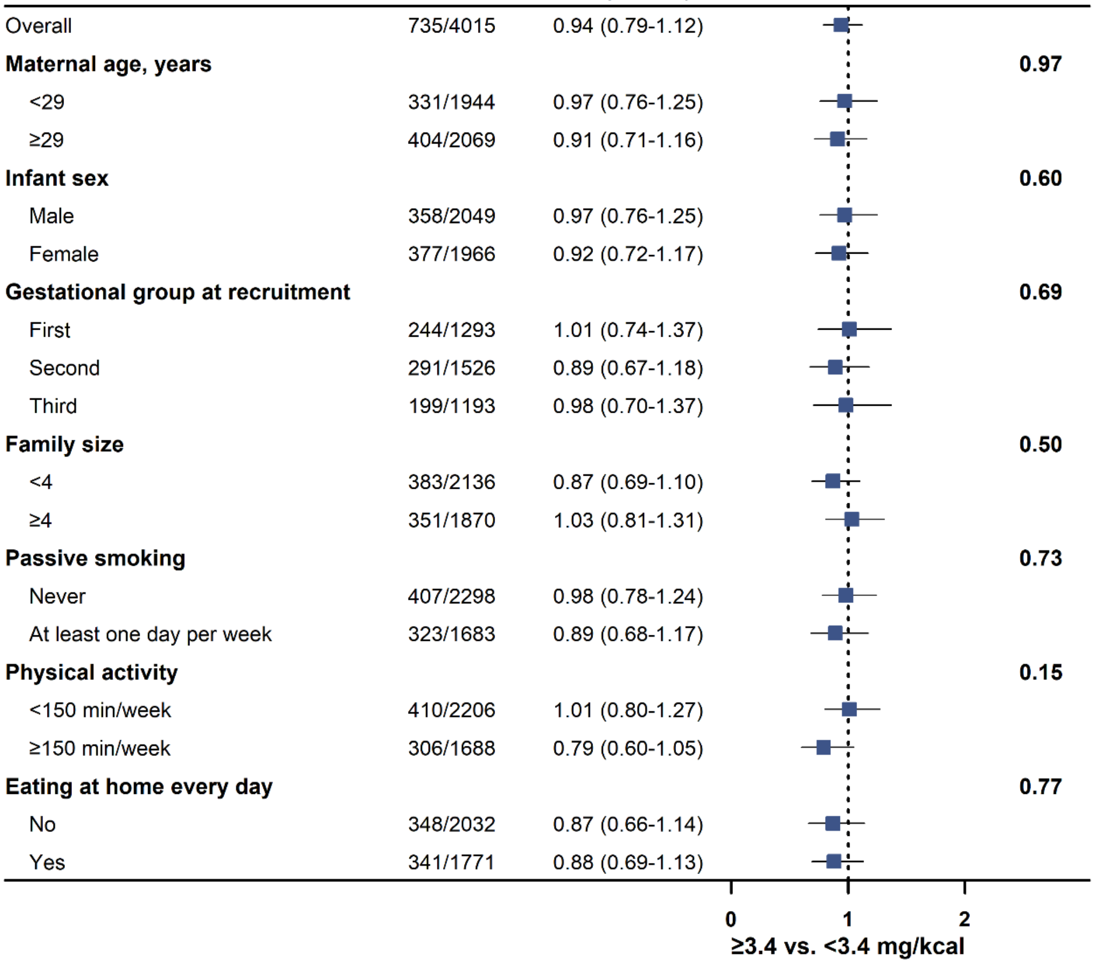

Figure S4. Forest plots for the association of salt density with the risk of large for gestational age in pre-specified subgroups.

ORs and 95% CI were calculated in logistic model adjusted for maternal age (continuous variables, in years), infant sex (male or female), maternal domicile (south China, north China, or surrounding area of Shanghai), gestational group at recruitment (first, second, or third trimester), maternal education (<13 years or ≥13 years), annual family income (<100 000, 100 000-350 000, or ≥350 000 yuan/year), pre-pregnancy BMI (<18.5, 18.5-23.9, or ≥24.0 kg/m^2), height (continuous variables, in cm), passive smoking during pregnancy

(never or ever), alcohol drinking (never or ever), physical activity (regular physical activity or not), and eating at home every day (yes or no). Salt density was categorized into two groups using the mean values (<3.4 (ref) and ≥3.4 mg/kcal) to minimize the small sample bias and improve the precision of estimates. N Cases/Total=number of cases and total participants in the study. OR=odds ratio. CI=confidence interval.
